# Social networks and quality of life among female breast cancer patients at Tikur Anbessa specialized hospital, Addis Ababa, Ethiopia 2019

**DOI:** 10.1186/s12905-020-00908-8

**Published:** 2020-03-11

**Authors:** Rahel Aberaraw, Abdisa Boka, Roza Teshome, Addisu Yeshambel

**Affiliations:** 1 Department of Oncology Nursing, Tikur Anbessa Specialized Hospital, Addis Ababa, Ethiopia; 2 School of Nursing and Midwifery, College of Health Science, Addis Ababa University, Addis Ababa, Ethiopia; 3 Department of Midwifery, College of Health Science and Medicine, Wolaita Sodo University, Wolaita Sodo, Ethiopia

**Keywords:** Social-networks, Quality of life, Breast cancer

## Abstract

**Background:**

Breast cancer is a major life-threatening global public health problem. It is the most common form of cancer in females in many developing countries including Ethiopia. Social networks could change the course of cancer and can influence the quality of life among breast cancer patients. Therefore, the purpose of this study was to assess social networks and quality of life among female breast cancer patients attending in Tikur Anbassa Specialized Hospital, Addis Ababa, Ethiopia 2019.

**Methods:**

An institutional-based cross-sectional study was conducted in Tikur Anbessa Specialized Hospital Addis Ababa, Ethiopia from March 1 to April 30/2019. A total of 214 female breast cancer patients were included Binary and multiple logistic regression was used to show the association of social networks and quality of life.

**Result:**

A total of 214 females with breast cancer were recruited with a mean age of 41.85. Participants who had children (AOR = 5, 95%CL: 1.3,21 COR = 6), and other relatives (AOR = 6, 95%CI: 1.2,30, COR = 7), were more likely to have good social networks. Participants who were not married (AOR = 0.02, 95%CI: 0.03, 0.28), had no parents living (AOR = 0.1, 95%CI: 0.02, 0.4), no close friends (AOR = 0.06, 95%CI: 0.01, 0.4), and no neighbors (AOR = 0.09, 95%CI: 0.03, 0.5) had poor social networks.

**Conclusion:**

The quality of life was relatively low and social support were found to be poor in women with breast cancer. Health-care providers in oncology departments need to focus on addressing the side effects of therapy and social networks which may help to improve the quality of life of females with breast cancer.

## Background

Breast cancer refers to cancer originating from breast tissue, most commonly from the inner lining of milk ducts or the lobules that supply the ducts with milk. It is the most common cancer and the principal cause of cancer-related deaths in females worldwide [1]. A breast cancer diagnosis not only affects the woman diagnosed but also has huge implications for those involved in their life [2]. Different studies have shown that the number of patients with breast cancer is rising sharply in recent years. Currently, the problem of breast cancer is likely to grow greatly in Africa [3]. Its burden has become a major public health problem in developing regions, as the incidence rate is rising in these regions of the world [3].

Annually in Ethiopia, around 60,000 new cases of breast cancer are diagnosed [4]. The Addis Ababa Cancer registry reports that breast cancer was the commonest cancer which accounts for 33% of all female cancer cases and 23% of all cancers [5]. The increase in number of cases from year to year is due to the increase in the awareness of women’s on the disease, its prognosis and early screening and diagnosis [6, 7] More than 50% of breast cancer occurs in premenopausal women, aged younger than 40 years and/or with stage 3 disease [7] or age of the women ranged from 20 to 88 years (median age 43.0 years) [6] due to women’s poor health seeking behaviors on quality of medical and nursing care, having children in their young age, low socio-economic status of females to cover needs and expenses associated with cancer and poor awareness breast cancer symptoms, prevention mechanisms, risk factors, and treatment options. Deaths of females from breast cancer during their most productive years could result in tragedy for families, food insecurity and children’s withdrawal from school, an increased work burden on children and loss of assets [8].

Social networks defined the network of social relationships that surround an individual and the characteristics of those bonds [9]. The most commonly examined aspect of social networks with regard to breast cancer outcomes has been social network size, i.e. the number of network members [10]. It is well established that larger social networks predict lower overall mortality in healthy populations [11]. Studies have initiated that larger networks (i.e. greater social integration) are associated with better survival [12, 13] and better quality of life after breast cancer. Social networks might impact cancer outcomes by influencing stage at detection or progression by affecting treatment decisions [12].

In a meta-analysis of 87 papers, larger social networks were found to be meaningfully connected with lower cancer mortality [14]. In other studies, larger networks were associated with increased quality of life [15]. A recent meta-analysis combining data from 87 studies of social networks and cancer consequences reported stronger inverse associations through cancer mortality among breast cancer survivors compared with other cancer sites [14].

Breast cancer is a worldwide problem, with 1.7 million new cases being diagnosed per year [16]. In a study conducted in Tikur Anbessa specialized hospital, breast cancer accounts for 29.4% of cancer cases followed by cancer of the cervix 26.3% [17]. Among cancer survivors, social networks have been related to improved quality of life [15]. In a Nurses’ Health Study (NHS) of 2835 females by any stage breast cancer, Kroenke and colleagues found that a socially isolated female was twice as likely to die of their breast cancer as socially integrated women [13]. One study has discovered that females with few social connections had a 43% higher risk of breast cancer returning, compared to well-connected females.. Likewise, the isolated female was 64% more likely to die from breast cancer and 69% more possible to die of any cause during the the study, compared to their complements with many social bonds [8].

A meta-analysis of 87 studies summarizing the literature on the association between social networks and cancer survival stated that having larger social networks and being married were connected with declines in risk ratios for mortality of 20, and 12%, respectively [14] and other literature supported that important relations of social network size and quality of life outcomes are significant mechanisms through which naturally occurring networks influence quality of life outcomes after a breast cancer diagnosis [15].

Socially-isolated individuals are less able to buffer the impact of health stressors than others and consequently are at greater risk of adverse health effects such as poor quality of life (QOL), illness or death [18]. The impact of social networks and quality of life has not been well characterized among Ethiopian breast cancer patients. Therefore, this study aimed to assess social networks and quality of life among female breast cancer patients in Addis Ababa, Ethiopia.

## Methods

### Study area and setting

The study was conducted at the Oncology center, TASH, located in Addis Ababa. Addis Ababa is the capital and the largest in Ethiopia. Tikur Anbassa Specialized Hospital (TASH) is a large government-owned referral teaching hospital, located in Kirkos sub-city under the administration of Addis Ababa University, College of Health Sciences. The oncology center at the Hospital is the only referral center in the country.

### Study design and period

An institutional-based cross-sectional study was conducted from March 1 to April 30/ 2019.

### Source population

All breast cancer patients being evaluated and treated in oncology units were considered as a source population.

### Study population

Those breast cancer patients visiting the hospital and being evaluated or treated at the oncology unit during data collection time and who met the eligibility criteria were invited.

### Inclusion criteria

All-female breast cancer patients who visited the hospital during the data collection were eligible for participation in the study.

### Exclusion criterion

Patients who are unable to respond and those who didn’t take chemotherapy treatment were excluded from the study.

### Sample size determination and sampling procedure

To describe the distribution of quality of life scores, social networks, and associated factors, the sample was calculated by using the prevalence of breast cancer patients of 14.8% [18], by adding 10% non- response rate, the total sample size was calculated to be 214.

According to the one-year record of female breast cancer, 8000 cases were seen in the oncology unit at TASH. Since the duration of the study was 4 weeks, the calculated flow within the 4 weeks was 667 and the required sample size was 214, “K” was 3, so every 3 eligible women were enrolled in the study during the data collection period. This included women who came to the hospital for initiation of treatment or follow-up during the data collection period.

### Dependent variables

Social networks among female breast cancer patients and Quality of life among female breast cancer patients.

### Independent variables

Socio-demographic (Age, educational status and religion), Socioeconomic (occupation and monthly income), Clinical factors: Body mass index (BMI), stage of the diseases, time since diagnosis and type of treatment, Lifestyle (smoking, alcohol intake and physical activity).

#### Operational definition

Social networks of the respondents were assessed using Cohen’s Social Network Index (SNI) which contains 12 items (at Cronbach’s Alpha coefficient of 0.72 was obtained) [19]. This index counts the number of social roles in which the respondent has regular contact, at least once every 2 weeks, with at least one person: (spouse, children, parents, partner’s parents, other relatives, close friends, religious, education, employment, neighbors, volunteer works, and other social groups). The maximum SNI score is 12. Three categories of social network diversity were formed based on the SNI score: SNI 0–3 represented a limited social network, 4–5 as a medium social network and SNI ≥6 as a diverse social network. A dichotomous variable was created for SNI, with a good social network defined as an SNI greater than or equal to 4 and a poor social network a score of less than 4 [20].

Quality of life was assessed by using functional scales (The alpha coefficient (internal consistency) for the total score was high (alpha = .90) symptom scales, and global health status scales [21]. The functional scale includes - Physical, Role, Cognitive, Emotional, Social Functioning, body image, sexual functioning, sexual enjoyment, and future perspective. Global health status assessed by two items. And symptom scales include - fatigue, nausea and vomiting, pain, dyspnea, insomnia, appetite loss, constipation, diarrhea, financial difficulty, systemic therapy side effects, breast symptoms, arm symptoms and upset by hair loss. Not affected quality of life: Participants who were scored 75 and above for functional and global health status scale and 25 and below for symptom scale. Affected quality of life: Participants who were scored below 75 for functional and global health status scale and above 25 for symptom scale [21].

#### Data collection tools

Data was collected by face to face interview using structured questionnaires that were adapted from SNI and Quality of life and global health status scales (The alpha coefficient (internal consistency) for the total score was high (alpha =0.94) [10, 21, 22]. The QLQ-C30 is the main questionnaire which is aimed to address health-related quality of life of cancer patients in general and it incorporates 30 items. Five functional scales (Physical, Role, Cognitive, Emotional and Social Functioning); three symptom scales (Fatigue, Pain and Nausea or Vomiting), a global health status scale, and a number of single items assessing additional symptoms commonly reported by cancer patients (dyspnea, loss of appetite, insomnia, constipation and diarrhea) and perceived financial impact of the disease. While, QLQ-BR23, which assesses the quality of life for breast cancer patients, has 23 items assessing disease symptoms, side effects of treatment, body image, sexual functioning and future perspective to predict the specific breast cancer related QoL predictors [23].

Thus, the 53 questions from EORTC (30 questions QLQ-C30 and 23 questions QLQ-BR23) were used to assess QOL. From the EORTC-C30 questions 1–15 were used to assess functional scale, from question 16–28 were used to assess symptom scale and the last 2 questions (29–30) were used to assess global health status scale. And from the EORTC-BR23 questions 1–8 were used to assess functional scales and 9–23 were used to assess symptom scales. The participants of the study were requested to select only one answer from (1- Not at all, 2- A little, 3- Quite a bit or 4- Very much) for the first 28 questions and they were asked to select one between the range from 1 (which means Very poor) to 7 (Excellent) in the EORTC QLQC30 items for global health status questions. When it comes to EORTC QLQ-BR23 questions, the participants were requested to select only one answer (1-Not at all, 2-A little, 3-Quite a bit or 4-Very much) for each question.

#### Data collection procedure

Six BSc nurses and two MSc supervisors were used for data collection. One day training was given for clarification of some terms and assessment tools. Ethical clearance was obtained from the institutional review board of Addis Ababa University, College of Health Sciences, School of Nursing and Midwifery. Informed written consent was gained from all study participants and for those under age 16, consent was obtained from their parents/guardians. After information was provided about the purpose of the study, non- invasiveness of the data collection procedure, confidentiality of the information and respondents were reassured that they would be anonymous (unnamed). Then respondents were given a chance to ask anything about the study and were free to refuse or stop at any moment during the study.

#### Data processing and analysis

Descriptive statistics were used to analyze demographic characteristics. Logistic regression models were used to evaluate associations between social networks, and quality of life. Bivariate and multivariate analysis with 95% CI was employed. Variables found to have a *P*-value< 0.2 in the binary logistic regression were entered into multivariate analysis and strength of association was declared at *P* value< 0.05.

## Result

### Socio-demographic characteristics of the participants

A total of 214 participants were included in this study. The mean age was 41.85 and the range of age was from 20 to 80 years. Most of the participants were Christian 142 (66.4%) followed by Muslim 41 (19.2%). Sixty-six (30.8%) of the participants were illiterate. From the total respondents, 104 (48.6%) were not employed outside the home. Ninety (42.1%) of the respondents had a monthly income greater than or equal to 2000 ETB (Ethiopian Birr). Regarding to time since diagnosis, more than two-third of participants, 155(72.5%) were diagnosed before 12 months. Among the total participants, 129 (60.3%) of them had received surgery with chemotherapy treatment (Table 1).

**Table 1:** Socio-demographic and socio-economic characteristics of the participants at TASH, Addis Ababa, Ethiopia 2019

Variable	Frequency *n* = 214	Percent
Age		
< 40	106	49.5
40–49	54	25.2
50–59	29	13.6
≥ 60	25	11.7
Religion		
Orthodox	136	63.6
Muslim	41	19.1
Protestant	31	14.5
Catholic	6	2.8
Educational status		
Illiterate	66	30.8
Grade1–8	38	17.8
Grade 9–12	64	29.9
College graduated	46	21.5
Occupation		
House wife	104	48.6
Governmental	44	20.6
Private	34	15.8
Student	3	1.4
Pension	29	13.6
Monthly income in ETB		
< 500	67	31.3
501–1000	27	12.6
1001–1500	20	9.3
1501–2000	10	4.7
≥ 2000	90	42.1
Smoking		
Current	9	4.2
Past	3	1.4
Never	202	94.4
Alcohol intake		
Current	2	0.9
Past	9	4.2
Never	203	94.9
Physical activity		
< 3	24	11.2
3–17	172	80.4
≥ 18	18	8.4
Type of treatment		
Chemotherapy	51	23.8
Surgery and chemotherapy	148	69.2
Surgery, chemotherapy and radiation therapy	15	7
Stage of diseases		
Stage 1	75	35
Stage 2	24	11.2
Stage 3	20	9.3
Stage 4	54	25.4
Recurrence	41	19.1
Time since diagnosis		
< 12 month	155	72.5
13–24 month	14	6.5
25–34 month	6	2.8
35–59 month	22	10.3
≥ 60 month	17	7.9

### Social networks characteristics of the participants

**Fig. 1:**
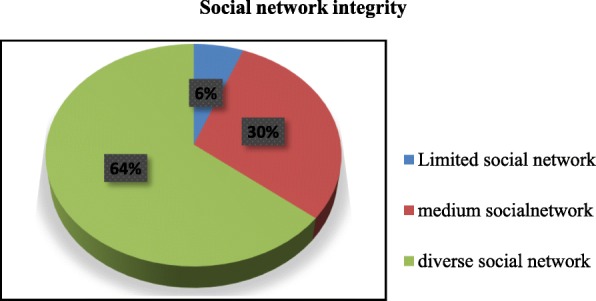
Social networks integrity among female breast cancer at TASH, Addis Ababa, Ethiopia 2019

Among total participants, 141 (65.9%) of them were married and 28 (13.1%) were divorced. Half of the respondents, 109 (50.9%) had one to three children whereas 45 (21%) had no children. Most of them, 196 (91.6%) had other relatives (other than parents, husband, and children) and 136 (63.6%) of them had close friends. The majority of the respondents, 199 (93%) participated in religious activity. Participants who were involved in regular volunteer work were 203 (94.9%) (Table 2). There were 12 items used to assess social networks of study participants among breast cancer patients. From 12 items, participants who had scored 0–3 were categorized as limited social networks. Participants who had scored 4–5 and ≥ 6 were categorized as medium social networks and diverse social network respectively. The logistic regression used the dichotomous social networks variable good vs poor. From total participants 13(6%), 65(30%) and 136(64%) had limited, medium and diverse social networks respectively (Fig. 1).

**Table 2:** Social networks characteristics of the participants among female breast cancer patients at TASH, Addis Ababa, Ethiopia 2019

Variable	Frequency *n* = 214	Percent
Marital status		
Married	141	65.9
Single	28	13.1
Divorced	28	13.1
Widowed	17	7.9
Number of children		
0	45	21
1–3	109	50.9
4–5	49	22.9
≥ 6	11	5.2
Parents living		
Neither	79	36.9
Mother	64	29.9
Father	15	7
Both	56	26.2
Partner’s parents Living		
Neither	131	61.2
Mother	39	18.2
Father	10	4.7
Both	34	15.9
Other relatives		
0	18	8.4
1–3	69	32.2
4–5	69	32.2
≥ 6	58	27.2
Close friends		
0	78	36.4
1–3	113	52.8
4–5	13	6.1
≥ 6	10	4.7
Belong to religious group		
Yes	199	93
No	15	7
Attend any class		
Yes	9	4.2
No	205	95.8
Employed full or part time		
No	149	69.6
Private	26	12.2
Governmental	39	18.2
Neighbors		
0	59	27.6
1–3	92	43
4–5	30	14
≥ 6	33	15.4
Volunteer work		
Yes	11	5.1
No	203	94.9
Belong to any group		
Yes	3	1.4
No	211	98.6

### Quality of life of the participants

Participants scored a global health status scale with a mean = 83.61 and SD = 20.9. From EORTC-C30 Functional scales, the best score was observed for social functioning that is a mean of 75.5 (SD = 26). Whereas, in the QLQ-BR23 functioning scales, the best score was observed for future perspective mean = 78 and SD = 33.6). Participants also had a low mean score [19] for sexual functioning (Table S1).

**Fig. 2:**
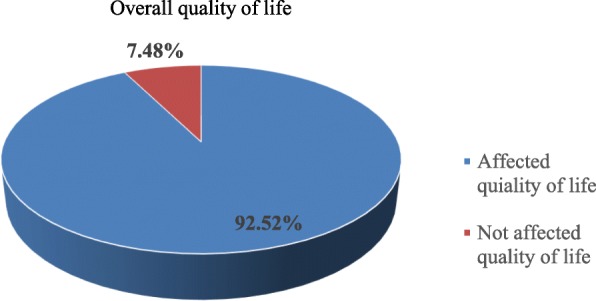
Overall quality of life among female breast cancer patients at TASH, Addis Ababa, Ethiopia 2019

To assess the quality of life of the participants, there are three subscales:- functional scale, symptom scales and global health status scale. Based on this, participants who scored 75 and above for functional and global health status scale and 25 and below for symptom scale classified as not affected the quality of life whereas, participants who scored below 75 for functional and global health status scale and above 25 for symptom scales classified as the affected quality of life. Among the total participants, 48(22.4%), 150(70.1%) and 192(89.7%) of them had affected QoL, in global health status scale, functional scales and symptom scales respectively. Participants who had not affected QoL were 16 (7.48%) (Fig. 2).

### Association of variables with social networks among female breast cancer patients

Among the total study participants, 132 (61.7%) had children. It was found that participants who had children were 5 times more likely to have good social networks than participants who had no children (AOR = 5, 95%CL: 1.3,21 COR = 6). From the total study participants, 189 (88.3%) of them had other relatives. It was found that participants who had other relatives were 6 times more likely to have good social networks than those who had no other relatives (AOR = 6, 95%CI: 1.2,30, COR = 7). However, participants who were not married (AOR = 0.02, 95%CI: 0.03, 0.28), had no parents living (AOR = 0.1, 95%CI: 0.02, 0.4), no close friends (AOR = 0.06, 95%CI: 0.01, 0.4), no job (AOR = 0.09, 95%CI: 0.02,0.46), did not belong to a church (AOR = 0.09, 95%CI:0.02,0.4) and had no relationship with neighbors (AOR = 0.09, 95%CI:0.03,0.5) had poor social networks (Table S2).

### Association of variables with quality of life among female breast cancer patients

Among the total study participants, 28(13.1%) were illiterate. It was found that participants who were illiterate were 3 times more likely to have affected QoL than educated (AOR = 3, 95%CI: 1.3, 6.9, COR = 4.8, *p*-value = 0.008). Among the total study participants, 45(21%) reported systematic therapy side effects. It was found that participants who had systematic therapy side effects were 3.8 times more likely to have affected QoL than who had no systemic therapy side effect (AOR = 3.8, 95%CI: 1.1,13, COR = 4, *p*-value = 0.035). Among the total participants, 45(21%) reported a loss of appetite. It was found that participants who had appetite loss were 3.5 times more likely to have affected QoL than those who did not report appetite loss (AOR = 3.5, 95%CI: 1.02,12 COR = 4, *p*-value = 0.047). In addition, those participants who had good social networks were 4.5 times ((AOR = 4.5, 95%CI: 1.30,15 COR = 6.4, *p*-value = 0.035)) more likely to have good quality of life when compared with women with poor social networks. (Table S3).

## Discussion

This study assessed social networks and QoL among female breast cancer patients in Ethiopia. The maximum social network score was ten out of a possible of 12, in this study. The finding is similar to a study done in New York, which was nine [8]. This similarity might be due to the use of the same tool/questionnaire to assess the social networks of the participants on breast cancer patients.

The average global health status score of study participants’ in this study was 83.6%. This result is consistent with a study from Nepal in which the global health status score (82.08) [24]. The similarity might be due to the use of similar study design in studies, similar study tools used during data collection and socio-demographic characteristics of study participants. However, the current finding is high compared to a study done in Addis Ababa that was (52.5) [21], the EORTC reference value mean score was (61.8 ± 24.6) [25] and in South India mean score was (77.93) [23]. This difference might be due to advancement of the stage of disease, type of treatment used and time since diagnosis.

In the EORTC functional scales scores, the role functioning was the lowest (23.8 ± 32.80) and the highest observed in social functioning (75.5 ± 26). The finding is comparable to the study conducted in Ethiopia with a mean score of 74.1 ± 28.5 [21]. The similarity might be due to the study design/ tool used in both studies, the socio-demographic similarity of study participants and study settings. But, the finding is lower comparing with the EORTC reference value of mean score (77) [25] and study conducted in South India mean score (87.7 ± 24.6) [23]. The difference might be due to educational level differences, study participant age difference, awareness about the disease’s consequence and stage of the diseases.

In QLQ-BR23 functioning scales, the highest mean score (78 ± 33.6) was observed in a future perspective scale. The finding is comparable in the study done in Addis Ababa Ethiopia’s mean score (82.1 ± 30.3) [21]. Whereas, the finding is greater than the study conducted in South India’s mean score was (72.62 ± 33.81) [23]. The difference might be due to participants’ psychological and social support received through informal ways such as family and in religious institutions.

In the QLQ-C30 symptom scales, a higher mean score (67.8 ± 22.8) was observed on pain scale. The finding was greater than the study conducted in South India with the mean score of (19.6 ± 26.64) [23], Ethiopia with the mean score of (46.0 ± 31.9) [21] and the EORTC reference value mean score (28.7 ± 28.7) [25]. This difference might be due to the availability of pain medication in today’s health setup, the proper use of pain medication by the patients, awareness about the importance of anti-pain medication and side effects by health care providers. In QLQ-BR23 symptom scales highest mean score (55.9 ± 17.7) was observed in systematic therapy side effects. This finding is greater than the study done in South India mean score (13.04 ± 11.93) [23] and in Addis Ababa Ethiopia mean score (34.6 ± 29.7) [21]. This alteration might be due to the type of treatment, stage of the diseases.

The study participants who were married, had children, and participated in religious activities in this study had good social networks compared with those who were unmarried, had no children and did not participate in religious activities. This finding is supported by the studies from the US [12], in California [10, 15]. This might be due to the fact that being married; having children and participating in religious activities will enhance the social, physical, and emotional and interconnections in their day to day activities. In addition to this, participants involved in religious activities were receiving more information regarding social networks than others.

Study participants who were illiterate were nearly 3 times more likely to have affected QoL than those who were educated. This finding is supported with a study conducted in Shanghai, China [26] more educated breast cancer patients had improved quality of life. The similarity might be due to the fact that educated individuals can easily understand about the side effects of treatment and proper management of treatment side effects and usage so that they can even communicate easily with their health care providers to improve their quality of life.

High monthly income in the current study was more likely to be associated with a good quality of life. This finding is in agreement with the study done in Shanghai, China [26] in which high monthly income was associated with good quality of life and study done in Addis Ababa [21], in which those who have reported that they didn’t have income, were less likely to have a good (unaffected) quality of life. This might be due to the fact that women with better income may better to access information regarding their health, nutrition, screening, and treatment and may have better quality life compared to low income women.

Participants who reported fatigue, nausea and vomiting, appetite loss and financial difficulty in this study were more likely to have affected QoL. Whereas, in a study conducted in Ethiopia [21] those who were having fatigue were less likely to have unaffected QoL and those who have no problem with nausea and vomiting, appetite loss and financial difficulties were more likely to have unaffected QoL. This similarity might be due to awareness of the patients on the stage of the diseases, type of treatment given for patients and availability of different cancer treatments in the hospital.

This study revealed that those study participants who had poor social networks were 6.4 times more likely to have an affected/lower quality of life. This is in line with two studies done in the US [15, 27, 28], in which larger social networks were associated with improved QoL after breast cancer. The comparison might be due to the fact that poor social networks which leads to social isolation were bring poor improvement the quality of life.

### Limitation of the study

The nature of this study was a cross-sectional one; it hinders the possibilities of assessing cause and effect associations. Furthermore, the design limits the progressive investigation of social networks and quality of life improvements following a series of intervention strategies. Results are also possibly subject to social desirability bias as the study outcome is self-reported.

## Conclusion

Based on the finding of this study above half of the total respondents having a diverse social network are a positive factor in determining a woman’s quality of life following a breast cancer diagnosis. Being married, having children, living parents, other relatives, close friends and belonging to a church were significantly associated with social networks. Whereas, education, monthly income, emotional functioning, role functioning, pain, fatigue, financial difficulty, systemic therapy side effect, and social networks were significantly associated with quality of life (QOL).

### Recommendations

Healthcare providers in specially working in oncology departments should teach community, attendants as well other stakeholders to address diverse social networks based on female needs and desires and the side effects of treatment, Strengthen awareness in collaboration with public medias about social networks, QoL and their association with breast cancer to improve social networks and the quality of life of females with breast cancer.

## Supplementary information

**Additional file 1: Table S1.** Quality of life of participants among female breast cancer patients at TASH, Addis Ababa, Ethiopia 2019. **Table S2.** Bivariate and multivariate logistic regression analysis of social networks and its explanatory variables among female breast cancer patients at TASH, Addis Ababa, Ethiopia 2019. **Table S3.** Bivariate and multivariate logistic regression analysis of Quality of life and its explanatory variables among female breast cancer patients at TASH, Addis Ababa, Ethiopia 2019.

## Data Availability

The data sets used and/or analyzed during the current study are available from the corresponding author on reasonable request.

## Abbreviations

AOR: Adjusted odds ratio
CI: Confidence interval
COR: Crude odds ratio
EORTC-QLQ-BR: European Organization on Research and Treatment of Cancer, Quality of life
Fig: Figure
NGO: Nongovernmental organization
QOL: Quality of life
SD: Standard deviation
TASH: Tikure-Anbessa Specialized Hospital

## References

[1] Meric F, Bernstam EV, Mirza NQ, Hunt KK, Ames FC, Ross MI (2002). Breast cancer on the world wide web: cross-sectional survey of quality of information and popularity of websites. Bmj.

[2] Murphy AR. Women with Breast Cancer and Their Significant Other: Snapshots of Doctoral Research at University College Cork; 2014.

[3] Pace LE, Shulman LN (2016). Breast cancer in sub-Saharan Africa: challenges and opportunities to reduce mortality. Oncologist.

[4] Fitzmaurice C, Dicker D, Pain A, Hamavid H, Moradi-Lakeh M, MacIntyre MF (2015). The global burden of cancer 2013. JAMA Oncol.

[5] Memirie ST, Habtemariam MK, Asefa M, Deressa BT, Abayneh G, Tsegaye B (2018). Estimates of Cancer incidence in Ethiopia in 2015 using population-based registry data. J Glob Oncol.

[6] Kantelhardt E (2014). Breast cancer survival in Ethiopia: a cohort study of 1,070 women. Int J Cancer.

[7] Abate S (2016). Trends of breast cancer in Ethiopia. Int J Cancer Res Mol Mech.

[8] Crookes DM, Shelton RC, Tehranifar P, Aycinena C, Gaffney AO, Koch P (2016). Social networks and social support for healthy eating among Latina breast cancer survivors: implications for social and behavioral interventions. J Cancer Surviv.

[9] Berkman LF, Glass T, Brissette I, Seeman TE (2000). From social integration to health: Durkheim in the new millennium☆. Soc Sci Med.

[10] Kroenke CH, Kwan ML, Neugut AI, Ergas IJ, Wright JD, Caan BJ (2013). Social networks, social support mechanisms, and quality of life after a breast cancer diagnosis. Breast Cancer Res Treat.

[11] Smith TW, Marsden P, Hout M, Kim J (2012). General social surveys. National Opinion Research Center.

[12] Beasley JM, Newcomb PA, Trentham-Dietz A, Hampton JM, Ceballos RM, Titus-Ernstoff L (2010). Social networks and survival after a breast cancer diagnosis. J Cancer Surviv.

[13] Kroenke CH, Kubzansky LD, Schernhammer ES, Holmes MD, Kawachi I (2006). Social networks, social support, and survival after a breast cancer diagnosis. J Clin Oncol.

[14] Pinquart M, Duberstein PR (2010). Associations of social networks with cancer mortality: a meta-analysis. Crit Rev Oncol Hematol.

[15] Kroenke CH, Quesenberry C, Kwan ML, Sweeney C, Castillo A, Caan BJ (2013). Social networks, social support, and burden in relationships, and mortality after breast cancer diagnosis in the life after breast Cancer epidemiology (LACE) study. Breast Cancer Res Treat.

[16] Mittra I (2011). Breast cancer screening in developing countries. Prev Med.

[17] Tadele N (2015). Evaluation of quality of life of adult cancer patients attending Tikur Anbessa specialized referral hospital, Addis Ababa Ethiopia. Ethiop J Health Sci.

[18] Hemmati A, Chung KSK. Social networks and quality of life: The national health interview survey. In: Advances in Social Networks Analysis and Mining (ASONAM), 2014 IEEE/ACM International Conference on: IEEE. China; 2014.

[19] Woldu M, Legese D, Abamecha F, Berha A (2017). The prevalence of Cancer and its associated risk factors among patients visiting oncology unit, Tikur Anbessa specialized hospital, Addis Ababa-Ethiopia. J Cancer Sci Ther.

[20] Cohen S, Doyle WJ, Skoner DP, Rabin BS, Gwaltney JM (1997). Social ties and susceptibility to the common cold. Jama.

[21] Meron MA. Assessing the Quality of life among patients with breast cancer at Tikur Anbassa Specialized Hospital, Addis Ababa, Ethiopia. 2016.

[22] Aung MN, Moolphate S, Aung TNN, Katonyoo C, Khamchai S, Wannakrairot P (2016). The social network index and its relation to later-life depression among the elderly aged≥ 80 years in northern Thailand. Clin Interv Aging.

[23] Dubashi B, Vidhubala E, Cyriac S, Sagar T (2010). Quality of life among young women with breast cancer: study from a tertiary cancer institute in South India. Indian J Cancer.

[24] Shrestha JS, Shresta A, Spkata A, Sharma R, Shrestha S, Shestha S (2017). Social support, quality of life and mental health status in breast cancer patients. Cancer Rep Rev.

[25] Scott N, Fayers P, Aaronson N, Bottomley A, de Graeff A, Groenvold M (2008). EORTC QLQ-C30. Reference values.

[26] Spatuzzi R, Vespa A, Lorenzi P, Miccinesi G, Ricciuti M, Cifarelli W (2016). Evaluation of social support, quality of life, and body image in women with breast cancer. Breast Care.

[27] Kroenke CH, Michael Y, Tindle H, Gage E, Chlebowski R, Garcia L (2012). Social networks, social support and burden in relationships, and mortality after breast cancer diagnosis. Breast Cancer Res Treat.

[28] Woldeamanuel YW, Girma B, Teklu AM (2013). Cancer in Ethiopia. Lancet Oncol.

