# Supplementary material for: Social networks and quality of life among female breast cancer patients at Tikur Anbessa specialized hospital, Addis Ababa, Ethiopia 2019
Source: BMC Womens Health. 2020 Mar 11;20:50. doi: 10.1186/s12905-020-00908-8 (PMC7065362; doi:10.1186/s12905-020-00908-8)
Supplement: Supplementary file 1 — Additional file 1: Table S1. Quality of life of participants among female breast cancer patients at TASH, Addis Ababa, Ethiopia 2019. Table S2. Bivariate and multivariate logistic regression analysis of social networks and its explanatory variables among female breast cancer patients at TASH, Addis Ababa, Ethiopia 2019. Table S3. Bivariate and multivariate logistic regression analysis of Quality of life and its explanatory variables among female breast cancer patients at TASH, Addis Ababa, Ethiopia 2019. [file 12905_2020_908_MOESM1_ESM.docx]

**Table S1: Quality of life of participants among female breast cancer patients at TASH, Addis Ababa, Ethiopia 2019.**

| **Scales** | **Mean ±SD** |
| --- | --- |
| **Global health status/QOL** | 83.61±20.9 |
| **EORTC QLQ-C30 functional scale** |  |
| Physical functioning | 60±21.8 |
| Role functioning | 23.8±32.8 |
| Emotional functioning | 59.1±38.7 |
| Cognitive functioning | 72.8±28.2 |
| Social functioning | 75.5±26 |
| **EORTC QLQ-C30 symptom scale** |  |
| Fatigue | 64.1± 23.5 |
| Nausea and vomiting | 30.7±28 |
| Pain | 67.8± 22.8 |
| Dyspnea | 41.3± 31.5 |
| Insomnia | 47.2±35.8 |
| Appetite loss | 59.3± 32.8 |
| Constipation | 14.8± 26 |
| Diarrhea | 14±29.3 |
| Financial difficulty | 63± 43 |
| **EORTC QLQ-BR23 functional scale** |  |
| Body image | 76.9± 33.5 |
| Sexual functioning | 20.1±20 |
| Sexual enjoyment | 35.6+25.4 |
| Future perspective | 78± 33.6 |
| **EORTC QLQ-BR23 symptom scale** |  |
| Systemic therapy side effect | 55.9±17.7 |
| Breast symptoms | 20.3± 21.6 |
| Arm symptoms | 20.4± 22.3 |
| Upset by hair loss | 15.6± 29 |

**Table S2: Bivariate and multivariate logistic regression analysis of social networks and its explanatory variables among female breast cancer patients at TASH, Addis Ababa, Ethiopia 2019.**

| Variables | Social- | Networks |  |  |
| --- | --- | --- | --- | --- |
|  | **Low n(%)** | **High n (%)** | **COR (95%CI)** | **AOR (95%CI)** |
| Education  illiterate  grade1-8  grade9-12  college | 8 (3.7)  1 (0.5)  1 (0.5)  3 (1.4) | 51 (23.8)  22 (10.3)  10 (4.7)  118 (55.1) | 0.16(0.04,0.64) *  0.56(0.05,5.62)  0.25(0.02,2.67)  1 | 0.28(0.54,30.34)  0.91(0.65,17.35)  0.95(0.03,9.43)  1 |
| Occupation housewife governmental  private  Student | 2 (0.9)  0 (0)  11 (5.1)  0 (0.0) | 185 (86.4)  4 (1.9)  1 0(4.7)  2 (0.9) | 1  0.01(0.05,1.57)  0.01(0.01,0.043) *  0.01(0.009,34.55) | 1  0.36(0.54,13.64)  0.93(0.65, 9.83)  0.9(0.65,38.34) |
| Alcohol intake  Past  Never | 10 (4.6)  3 (1.4) | 1 (0.5)  200 (93.5) | 0.002(0.00,0.02) *  1 | 0.017(0.01,20.15)  1 |
| Married  No  Yes | 12 (5.6)  1 (0.5) | 61 (28.5)  140 (65.4) | 0.036(0.001,0.28*  1 | 0.03(0.03,0.28) **  1 |
| Children  No  Yes | 10 (4.7)  3 (1.4) | 69 (32.2)  132 (61.7) | 1  6(1.6,23) * | 1  5(1.3,21) ** |
| Parents living  No  Yes | 10 (4.7)  3 (1.4) | 69 (32.2)  132 (61.7) | 0.15(1.7,23.9) *  1 | 0.1(0.02,0.4) **  1 |
| Other relatives  No  Yes | 4 (1.9)  9 (4.2) | 12 (5.6)  189 (88.3) | 1  7(1.8,26) * | 1  6(1.2,30) ** |
| Close friends  No  Yes | 4 (1.9)  9 (4.2) | 12 (5.6)  189 (88.3) | 0.14 (0.04,0.5) *  1 | 0.06(0.01,0.4) **  1 |
| Belong to church  No  Yes | 4 (1.9)  9 (4.2) | 12 (5.6)  189 (88.3) | 0.14 (0.04,0.5) *  1 | 0.09(0.02,0.4) **  1 |
| Job  No  Yes | 7 (3.3)  6 (2.8) | 50 (23.4)  151 (70.6) | 0.284 (0.09,0.9) *  1 | 0.09(0.02,0.46) **  1 |
| Neighbors  No  Yes | 7 (3.3)  6 (2.8) | 50 (23.4)  151 (70.6) | 0.284 (0.09,0.9) *  1 | 0.13(0.03,0.5) **  1 |

**Table S3: Bivariate and multivariate logistic regression analysis of Quality of life and its explanatory variables among female breast cancer patients at TASH, Addis Ababa, Ethiopia 2019.**

| **Variables** | **Quality of life** |  |  |  |
| --- | --- | --- | --- | --- |
|  | Not affected n (%) | Affected n (%) | COR (95%CI) | AOR (95%) |
| Education  Illiterate  Grade1-8  Grade9-12  College | 40(18.7)  19(8.9)  12(5.6)  95(44.4) | 28(13.1)  2(0.9)  4(1.9)  14(6.5) | 4.8(2.3,9.6) *  0.7(0.15,3.4)  2.3(0.64,8)  1 | 3(1.3,6.9) **  0.7(0.14,3.33)  2(0.7,10)  1 |
| Monthly income  <500  501-1000  1001-1500  1501-2000  >2000 | 31(14.5)  13(6.1)  4(1.9)  14(6.5)  104(48.6) | 23(10.7)  2(0.9)  3(1.4)  2(0.9)  18(8.4) | 4(2,8.9) *  0.9(0.25,4.28)  4.3(0.9,21)  0.8(0.2,3.9)  1 | 2.6(1.2,6.2) **  0.7(0.13,3.4)  2.5(0.47,13.7)  0.8(0.2,3.9)  1 |
| Emotional functioning  Affected  Not affected | 90(42.1)  76(35.5) | 39(18.2)  9(4.2) | 1  3.6(1.67,8) * | 1  2.5(1.1,6) ** |
| Cognitive functioning  Affected  Not affected | 72(33.6)  94(43.9) | 32(15)  16(7.5) | 2.6(1.3,5) *  1 | 2.3(1.1,3) **  1 |
| Fatigue  Affected  Not affected | 133(62.1)  33(15.4) | 46(21.5)  2(0.9) | 5.7(1.3,24) *  1 | 5.2(1.1,15) ** |
| Nausea/vomiting  Affected  Not affected | 99(46.3)  67(31.3) | 38(17.8)  10(4.7) | 2.6(1.2,5.5) *  1 | 2.3(1.1,5) ** |
| Pain  Affected  Not affected | 112(52.3)  54(25.2) | 42(19.6)  6(2.8) | 3.4(1.3,8.4) *  1 | 3(1.2,8) **  1 |
| Insomnia  Affected  Not affected | 101(47.2)  65(30.4) | 41(19.2)  7(3.3) | 3.8(1.6,9) *  1 | 3(1.2,7) **  1 |
| Appetite loss  Affected  Not affected | 130(60.7)  36(16.8) | 45(21)  3(1.4) | 4(1.2,14) *  1 | 3.5(1.02,12) **  1 |
| Financial difficulty  Affected  Not affected | 118(55.1)  48(22.4) | 42(19.6)  6(2.8) | 2.8(1.1,7) *  1 | 2.6(1.01,6.8) **  1 |
| Systemic therapy side effect  Affected  Not affected | 130(60.7)  36(16.8) | 45(21)  3(1.4) | 4(1.2,14) *  1 | 3.8(1.1,13) **  1 |
| Social networks  Poor  Good | 5(2.3)  161(75.2) | 8(3.7)  40(18.7) | 6.4(1.9,20) *  1 | 4.5(1.3,15) **  1 |
|  |  |  |  |  |
